# Evaluating the association between migraine treatments and tinnitus: Insights from the US Food and Drug Administration adverse event reporting system

**DOI:** 10.1371/journal.pone.0330493

**Published:** 2025-08-20

**Authors:** Yun Kim, Yeabin Yook, Su-jin Rhee

**Affiliations:** 1 College of Pharmacy, Daegu Catholic University, Gyeongsan, Republic of Korea; 2 Department of Pharmacy, Wonkwang University College of Pharmacy, Iksan, Republic of Korea; 3 Institute of Pharmaceutical Research, Wonkwang University College of Pharmacy, Iksan, Republic of Korea; The Affiliated Hospital of Zhejiang Chinese Medical University, CHINA

## Abstract

Tinnitus, a distressing condition that can significantly impair quality of life, has been associated with several medications, including triptans. This study aimed to explore the relationship between tinnitus and specific migraine treatments, focusing on triptans and calcitonin gene-related peptide (CGRP) inhibitors. Data from the FDA Adverse Event Reporting System (FAERS) through the third quarter of 2023 were analyzed to calculate proportional reporting ratios (PRR) and reporting odds ratios (ROR) for migraine treatments, specifically triptans and CGRP inhibitors. Positive tinnitus signals were identified when PRRs or RORs were greater than 2.0, the lower bound of the 95% confidence interval exceeded 1.0, and at least three cases were reported. Intra- and inter-class analyses were conducted to compare tinnitus reports among individual drugs and drug classes. Among 47,615 tinnitus-related adverse events, 345 were associated with CGRP inhibitors and 183 with triptans. Positive tinnitus signals were observed for several CGRP inhibitors, except eptinezumab and atogepant, and for all triptans except sumatriptan. Inter-class analysis revealed no significant differences between triptans and CGRP inhibitors. However, intra-class analysis identified naratriptan, almotriptan, and frovatriptan as having notable tinnitus signals among triptans, while CGRP inhibitors did not exhibit strong signals for any specific drug. Using real-world data from FAERS and pharmacovigilance methods, this study identified tinnitus signals related to migraine treatments, particularly among certain triptans. These findings provide preliminary evidence for further investigation into the relationship between migraine medications and tinnitus.

## Introduction

Tinnitus, often described as the perception of sound in the absence of any external auditory stimulus, is a prevalent and distressing condition affecting millions of individuals worldwide [1–3]. From a faint ringing to a persistent buzzing or roaring, tinnitus manifests in various forms and intensities, profoundly impacting the daily lives of those affected. Tinnitus is bothersome and persistent, sometimes lasting for 6 months or longer, and it often negatively impacts patient’s quality of life. While most patients are less severely affected, some may experience anxiety, depression, and extreme life changes [4]. Despite its prevalence, the underlying mechanisms of tinnitus remain complex and multifaceted, posing significant challenges for effective management and treatment [1,5]. To date, no medications have been specifically approved to treat tinnitus by the US Food and Drug Administration (FDA) [6,7].

While the exact causes of tinnitus are not fully understood, it has been associated with factors such as exposure to loud noise, hearing loss, blockage of the ear canal by earwax or by fluid from an ear infection, head or neck injuries damaging structures of the ear. Tinnitus can also be a side effect of taking certain medications, especially if they are taken at high doses. Medications associated with tinnitus include non-steroidal anti-inflammatory drugs (e.g., ibuprofen, naproxen, and aspirin), certain antibiotics, anti-cancer drugs, and anti-malaria medications [8,9]. Triptans, a class of drugs commonly prescribed for the relief of migraine headaches, have also been occasionally associated with reports of tinnitus onset or exacerbation [10–13]. While the precise mechanisms underlying this potential association are not fully understood, the recognition of such adverse event reports highlights the need to investigate the relationship between migraine treatments and tinnitus.

Various therapies are available for the treatment and prevention of migraines, including onabotulinumtoxinA, beta-blockers like propranolol and timolol, and anticonvulsants such as topiramate, divalproex sodium, valproate sodium, and valproic acid. Additionally, serotonin receptor agonists, including triptans (5-HT_1B/1D_) and lasmiditan (5-HT_1F_), as well as the nonsteroidal anti-inflammatory drug (NSAID) celecoxib, have received approval for acute or episodic migraine treatment. Current clinical guidelines often recommend triptans as a prior treatment option [14]. Furthermore, the landscape of migraine therapeutics has evolved in recent years with the emergence of calcitonin gene-related peptide (CGRP) inhibitors. These novel medications represent a promising avenue for migraine management, offering new possibilities for patients who have experienced limited relief or intolerable side effects with existing treatments [15]. Nevertheless, like with any novel class of drugs, it is crucial to conduct a comprehensive evaluation of their safety profile, including the potential risk of adverse events. For instance, while the occurrence of tinnitus as an adverse reaction hasn’t been documented in the Food and Drug Administration (FDA) labeling of CGRP inhibitors thus far, a prior study analyzing disproportionality signals through the FDA’s voluntary adverse event reporting system (FAERS) identified tinnitus as a notable signal for erenumab [16].

The FAERS is a pivotal resource for post-marketing safety surveillance of approved drugs, enabling the accumulation and management of adverse event reports submitted voluntarily by consumers, pharmaceutical companies, healthcare professionals, and others involved in drug usage. FAERS compiles adverse events and medication errors reported to the FDA, and presents them in a user-friendly and interactive format through its public dashboard. The accessibility and comprehensiveness of FAERS data have spurred a surge in research endeavors aimed at evaluating the potential for adverse reactions associated with medications and uncovering novel adverse event insights previously undisclosed, thereby driving advancements in pharmacovigilance and drug safety [17,18].

Against this background, our study seeks to explore the intricate interplay between tinnitus and specific migraine treatments, with a particular focus on triptans and CGRP inhibitors. Utilizing the vast dataset accessible via the FAERS database, we aim to elucidate patterns of tinnitus occurrence associated with these medications. Our investigation aims not only to quantify tinnitus prevalence in patients receiving these treatments but also to compare different and within-class drugs to evaluate risk and better understand potential causes of tinnitus associated with their use.

## Materials and methods

### Data source

The FAERS adverse event reports from the first quarter of 1968 through the third quarter of 2023 were extracted, with a focus on serotonin receptor agonists (e.g., triptans and lasmiditan) and CGRP inhibitors. Additionally, FDA-approved migraine treatments, including onabotulinumtoxinA, beta-blockers (e.g., propranolol and timolol), anticonvulsants (e.g., topiramate and valproates), and celecoxib, were also included in the analysis. Nonproprietary medication names were utilized to evaluate individual medications; combination products were excluded to ensure clarity and precision. Tinnitus cases were identified using the Medical Dictionary for Regulatory Activities preferred term “tinnitus” [19]. Only adverse events reported under this specific preferred term were included to ensure methodological consistency [20]. The data used in this study were accessed in November 2023 from the publicly available FDA website. As this study used publicly available, anonymized data from the FAERS, no identifiable personal information was accessed. Therefore, institutional review board approval and informed consent were not required.

### Disproportionality analysis

The association between tinnitus and migraine treatments was assessed using a case/non-case approach. Tinnitus events reported for a suspect drug were considered cases, while other events were considered non-cases. Proportional Reporting Ratio (PRR) and Reporting Odds Ratio (ROR) were calculated to measure disproportionality, comparing tinnitus cases associated with each medication to those reported with all other drugs. PRR and ROR calculations were conducted using Microsoft Excel 2019. The PRR formula evaluated the ratio of reports for tinnitus with the medication of interest to all other medications, while the ROR compared the odds of reporting tinnitus with the medication to all other medications [18,21]. Additional analyses were performed focusing on specific subgroups of tinnitus reports restricted to migraine prophylaxis, migraine, or headache as a reason for use.

Significant signal indicators were determined based on predefined thresholds, with a PRR or ROR > 2.0 and the lower limit of a 95% confidence interval >1.0 considered positive signals. Additionally, signals required a minimum of three reported cases to ensure reliability [22,23]. Any identified signals were thoroughly investigated for clinical relevance and patient safety implications. Additionally, the FDA labels of each medication were scrutinized to determine if tinnitus had been reported as an adverse reaction. This step aimed to offer comprehensive insight into potential associations between tinnitus and the specified migraine treatments, and to assess the consistency of the analysis results with existing drug characteristic information.

### Intra- and inter-class analysis

To comprehensively understand tinnitus reporting patterns among migraine medications, both intra-class and inter-class investigations were conducted. Intra-class analyses were performed within each of the serotonin receptor agonist and CGRP inhibitor classes to delve deeper into the association of individual drugs within each class with tinnitus. Additionally, for inter-class analysis, each migraine drug treatment class was compared to either triptans or CGRP inhibitors as references, respectively, using PRR and ROR calculations. This approach was intended to account for potential confounding factors and provide insights into the relative tinnitus reporting among different classes of migraine treatments.

## Results

### Data description

A total of 81,147 adverse events were reported for CGRP inhibitors, and 44,105 adverse events were reported for serotonin receptor agonists (Table 1). Among the 47,615 adverse events related to tinnitus, 345 were associated with CGRP inhibitors, and 187 were associated with serotonin receptor agonists (183 with triptans and 4 with lasmiditan). For most drugs, the incidence rate of tinnitus compared to the total number of events was less than 1%, ranging from 0.3% to 0.6% for CGRP inhibitors, and 0.3% to 3.2% for serotonin receptor agonists. However, certain triptans, such as frovatriptan, naratriptan, and almotriptan, showed higher incidence rates ranging from 1.9% to 3.2%.

**Table 1:** Descriptive statistics for adverse events and tinnitus cases for each migraine drug reported in FAERS database.

Pharmacologic class		Drug name	Indication unrestricted^a^		PRR (95% CI)	ROR (95% CI)	Indication restricted^a^		PRR (95% CI)	ROR (95% CI)	Tinnitus on drug label
			Total events	Tinnitus cases			Total events	Tinnitus cases			
CGRP inhibitors	CGRP antagonists	Fremanezumab^c^	5835	36	3.51 (2.54–4.87)	3.53 (2.54–4.90)	2660	28	5.99 (4.15–8.66)	6.05 (4.17-8.78)	N
		Galcanezumab^c^	20390	78	2.18 (1.75–2.72)	2.18 (1.75–2.73)	10216	56	3.12 (2.40–4.05)	3.13 (2.41-4.08)	N
		Eptinezumab	2862	6	1.19 (0.54–2.65)	1.19 (0.54–2.66)	2190	4	1.04 (0.39–2.77)	1.04 (0.39-2.77)	N
	CGRP receptor antagonists	Erenumab^c^	41117	187	2.59 (2.25–2.99)	2.60 (2.25–3.00)	25341	136	3.06 (2.59–3.62)	3.07 (2.59-3.64)	N
		Rimegepant	6493	22	1.93 (1.27–2.93)	1.93 (1.27–2.94)	3634	17	2.66 (1.66–4.28)	2.67 (1.66-4.30)	N
		Ubrogepant^c^	1663	8	2.74 (1.37–5.47)	2.75 (1.37–5.50)	1024	5	2.78 (1.16–6.66)	2.79 (1.16-6.71)	N
		Atogepant	2787	8	1.63 (0.82–3.26)	1.64 (0.82–3.27)	1115	4	2.04 (0.77–5.43)	2.05 (0.77-5.46)	N
Serotonin receptor agonists	Triptans	Sumatriptan	30573	78	1.45 (1.16–1.81)	1.45 (1.16–1.82)	16867	61	2.06 (1.60–2.65)	2.06 (1.60-2.65)	N
		Zolmitriptan^c^	3402	20	3.35 (2.16–5.18)	3.36 (2.16–5.22)	2089	19	5.18 (3.31–8.10)	5.22 (3.32-8.19)	Y
		Rizatriptan^c^	3252	25	4.38 (2.96–6.47)	4.40 (2.97–6.53)	2282	20	4.99 (3.22–7.72)	5.02 (3.23-7.8)	Y
		Naratriptan^c^	991	20	11.49 (7.44–17.73)	11.71 (7.52–18.23)	759	20	15.00 (9.73–23.12)	15.38 (9.86-23.98)	N
		Almotriptan^c^	574	11	10.91 (6.07–19.59)	11.10 (6.11–20.16)	428	11	14.63 (8.16–26.22)	14.99 (8.24-27.28)	Y
		Frovatriptan^c^	436	14	18.28 (10.92–30.6)	18.85 (11.07–32.11)	367	14	21.71 (12.99–36.3)	22.54 (13.21-38.45)	Y
		Eletriptan^c^	4042	15	2.11 (1.27–3.50)	2.12 (1.27–3.51)	2729	13	2.71 (1.58–4.66)	2.72 (1.58-4.69)	N
	Ditan	Lasmiditan^c^	835	4	2.73 (1.03–7.25)	2.73 (1.02–7.30)	445	4	5.12 (1.93–13.57)	5.15 (1.93-13.79)	N
Acetylcholine release inhibitor		OnabotulinumtoxinA	56687	194	1.95 (1.70-2.25)	1.95 (1.70–2.25)	6575	40	3.46 (2.54–4.72)	3.48 (2.55–4.75)	Y
Beta-blockers		Propranolol^c^	23914	199	4.75 (4.14-5.46)	4.78 (4.16–5.50)	2064	30	8.28 (5.80–11.81)	8.38 (5.85–12.02)	N
		Timolol	13047	79	3.45 (2.77-4.30)	3.46 (2.78–4.32)	24	–	NA	NA	Y
Anticonvulsants		Topiramate^c^	34630	193	3.18 (2.76-3.66)	3.19 (2.77–3.68)	8934	113	7.21 (6.00–8.66)	7.29 (6.06–8.78)	N
		Valproates^b^	80700	199	1.40 (1.22-1.61)	1.41 (1.22–1.62)	1377	10	4.13 (2.23–7.67)	4.16 (2.23–7.74)	Y
NSAID		Celecoxib	68663	430	3.59 (3.26-3.94)	3.60 (3.28–3.96)	646	3	2.64 (0.85–8.17)	2.65 (0.85–8.24)	Y

PRR, proportional reporting ratio; ROR, reporting odds ratio; CI, confidence interval; CGRP, calcitonin gene-related peptide; FAERS, United States Food and Drug Administration Adverse Event Reporting System; NA, not applicable.

^a^“Indication restricted” refers to cases where the medications were specifically prescribed for the treatment of migraine prophylaxis, migraine, or headache, while “indication unrestricted” includes cases where the medications were used for various indications, not limited to migraine or headache.

^b^Valproates include divalproex sodium, valproate sodium, and valproic acid.

^c^Drugs meeting significance criteria in both PRR and ROR regardless of indication restrictions. Significance is determined when there are at least 3 cases reported, the point estimate of PRR and ROR is 2 or higher, and the lower limit of the confidence interval of the point estimate is 1 or higher.

Among tinnitus cases, 46.7% of those associated with CGRP inhibitors and 69.4% of those associated with serotonin receptor agonists were classified as serious. Notably, fremanezumab and erenumab among CGRP inhibitors, and all triptan drugs except for zolmitriptan showed high rates (>50%) of serious cases. The majority of tinnitus adverse events related to CGRP inhibitors and serotonin receptor agonists were reported in women (70.1% and 75.4%, respectively). The reported age range for tinnitus associated with CGRP inhibitors was 21–77 years, while for serotonin receptor agonists (triptans), it was 15–81 years.

### Drug label and disproportionality signals

None of the FDA drug labels for CGRP inhibitors mentioned tinnitus occurrences. However, for triptans, some drugs such as zolmitriptan, rizatriptan, almotriptan, and frovatriptan were suggested to have possible tinnitus side effects, while this possibility was not addressed for lasmiditan. Other migraine drugs such as onabotulinumtoxinA, valproates, and celecoxib acknowledged potential tinnitus risks in their labels. More than three cases of tinnitus were reported for all CGRP inhibitors and serotonin receptor agonists. Positive tinnitus signals were identified for specific CGRP inhibitors (i.e., fremanezumab, galcanezumab, erenumab, and ubrogepant) and all serotonin receptor agonists except for sumatriptan. This significant trend remained consistent even when analyzing cases restricted to migraine-related indications (Table 1).

### Intra- and inter-class comparison

**Fig 1:**
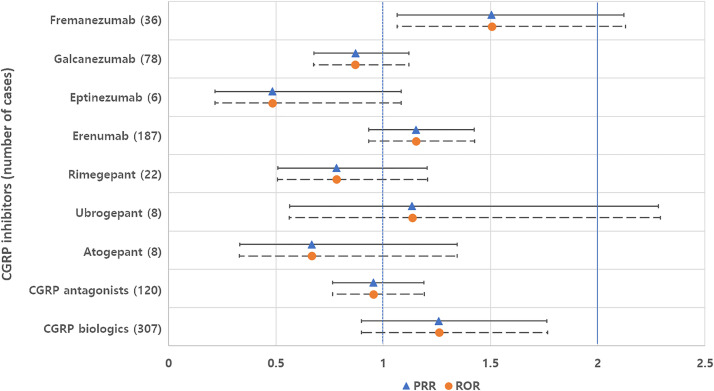
Proportional reporting ratios (PRR) and reporting odds ratios (ROR), each accompanied by their 95% confidence intervals, contrast tinnitus cases associated with individual calcitonin gene-related peptide (CGRP) inhibitors (or the broader CGRP drug class) against other drugs in the same class. CGRP antagonists include fremanezumab, galcanezumab, and eptinezumab. CGRP biologics include erenumab, fremanezumab, galcanezumab, and eptinezumab.

**Fig 2:**
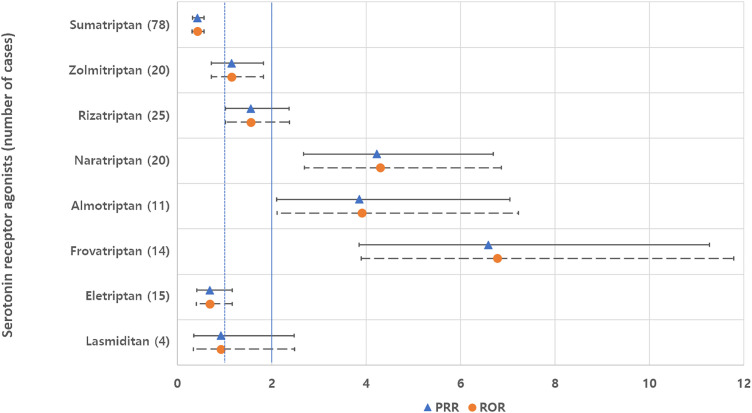
Proportional reporting ratios (PRR) and reporting odds ratios (ROR), each accompanied by their 95% confidence intervals, contrast tinnitus cases associated with individual serotonin receptor agonists against other drugs in the same class.

Individual drugs within the CGRP inhibitor class showed non-significant values for both PRR and ROR compared to other CGRP inhibitors (Fig 1). Conversely, naratriptan, almotriptan, and frovatriptan within the serotonin receptor agonist class showed significantly higher likelihoods of developing tinnitus compared to other drugs in the class (Fig 2).

**Fig 3:**
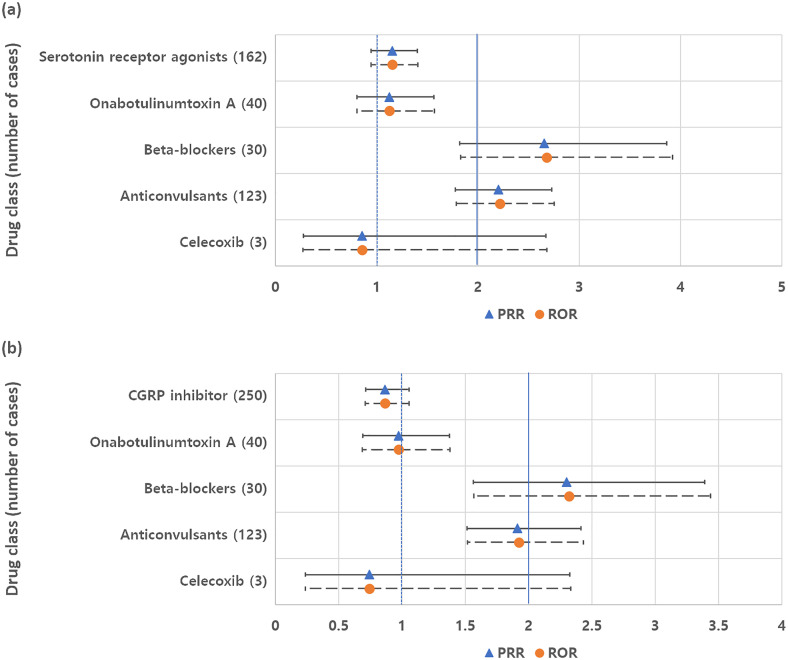
Inter-class analysis of migraine drugs with indications restricted to migraine prophylaxis, migraine, or headache. Proportional reporting ratios (PRR) and reporting odds ratios (ROR), along with their respective 95% confidence intervals, contrast tinnitus cases associated with each migraine drug class against (a) CGRP inhibitors and (b) serotonin receptor agonists. CGRP stands for calcitonin gene-related peptide.

In the inter-class analysis, no significant signal differences were found between serotonin receptor agonists and CGRP inhibitors. However, beta-blockers (i.e., propranolol) and anticonvulsants (i.e., topiramate and valproates) showed higher likelihoods of causing tinnitus when compared to CGRP inhibitors or serotonin receptor agonists (Fig 3). However, in the analysis that did not restrict indications, this trend disappeared in the case of anticonvulsants, and the significance of beta-blockers also decreased (S1 Fig).

## Discussion

This study investigated the occurrence of tinnitus as a potential adverse drug reaction among medications commonly used to treat migraine, utilizing real-world data from the FAERS. Through comprehensive comparisons and evaluations of individual drugs and drug classes, we identified medications associated with either a lower or higher risk of tinnitus being reported. Our findings provide preliminary insights into the safety profiles of triptans and CGRP inhibitors, and uncover potential adverse events not previously addressed in drug labels. This study is significant because no previous research has comprehensively compared the risk of tinnitus across various migraine treatments.

Tinnitus, a bothersome symptom with no established treatment due to its unclear etiology and pathology, poses significant disruptions to daily life. Previous studies have highlighted a significant relationship between tinnitus and headache, suggesting a possible shared pathophysiological basis. Specifically, findings indicate that headaches often precede tinnitus onset, hinting at a potential temporal relationship between the two conditions. However, the exact causality or sequence between headaches and tinnitus remains elusive [24–28]. Prior research has reported improvements in tinnitus symptoms through the regulation of migraine triggers [29]. Furthermore, one study found that the severity of tinnitus and headache coincided in nearly half of the participants; in other words, when head pain intensified, their tinnitus followed suit, and vice versa [24]. Considering these insights collectively, the significant signal of tinnitus observed across most migraine treatments in our study suggests two plausible interpretations: firstly, the possibility of tinnitus manifestation as an adverse drug reaction, and secondly, the potential association between migraine and tinnitus occurrence, given that migraine sufferers, who primarily utilized these medications, showed a higher incidence of tinnitus.

Despite the emergence of a significant tinnitus signal, variations exist among drugs within each class of migraine treatments regarding the risk of tinnitus occurrence. For instance, among triptan drugs, sumatriptan and eletriptan demonstrated a relatively low likelihood of tinnitus development in our study, with no mention of tinnitus adverse reactions on their labels. Conversely, frovatriptan, naratriptan, and almotriptan exhibited a heightened risk of tinnitus compared to other triptans. Interestingly, previous study comparing the efficacy and safety of triptan drugs revealed that frovatriptan and naratriptan displayed relatively lower efficacy and slower onset of action when compared to Sumatriptan 100 mg as the standard dose [30,31]. Conversely, eletriptan, rizatriptan, and zolmitriptan, which showed relatively low signal indicators for tinnitus in our study, were associated with notable therapeutic effects among triptans. Specifically, eletriptan and rizatriptan demonstrated the highest pain-free rates at 2 hours among oral triptans, with eletriptan also exhibiting the highest 24-hour sustained pain-free rate. Additionally, a hierarchy of treatment effects has been suggested with eletriptan, almotriptan, and zolmitriptan ranked in order [32]. One meta-analysis study indicated that all oral triptans were effective and well-tolerated, with rizatriptan, eletriptan, and almotriptan providing the highest likelihood of consistent treatment success [33]. This raises the possibility that tinnitus occurrence might be related to differences in the effectiveness of migraine treatments, rather than being solely an adverse drug reaction. However, the case of almotriptan presents a paradox, as it is recognized for its consistent treatment success and efficacy, yet shows a high incidence of tinnitus [32,33]. This suggests a multifaceted relationship between migraine and tinnitus, implying that factors beyond drug efficacy may influence the observed results.

In light of these aspects, despite the possibility that tinnitus may simply be a concomitant symptom of migraine, further research is needed to explore these complexities and determine whether certain medications (such as almotriptan, naratriptan, and frovatriptan, as highlighted in our study) pose a higher risk for tinnitus in migraine patients. In particular, prospective observational studies or randomized controlled trials that explicitly monitor tinnitus as an adverse event would be valuable to better understand potential causal relationships. To our knowledge, tinnitus has rarely been reported as a pre-specified safety outcome in randomized controlled trials of migraine treatments, which limits the ability to assess this risk based on clinical trial data alone. Additionally, preclinical studies evaluating auditory effects of migraine medications may help elucidate the biological mechanisms underlying the tinnitus signals observed in pharmacovigilance data.

Regarding CGRP inhibitors, there remains uncertainty about the possibility of tinnitus occurrence, and their drug labels do not list tinnitus as an adverse reaction. However, our study highlighted that some CGRP inhibitors, such as fremanezumab, galcanezumab, and erenumab, are more likely associated with tinnitus occurrence. This finding is consistent with a previous study which analyzed FAERS data and suggested a significant tinnitus signal with erenumab, while the study suggested a potential coexistence of migraine and tinnitus, rather than interpreting it solely as an adverse drug reaction [16]. Nevertheless, given the variability in tinnitus occurrence among CGRP inhibitors, careful selection of medications is recommended, especially in patients with pre-existing tinnitus.

For other medications such as propranolol, topiramate, and valproates, our analysis showed a higher risk of tinnitus when these drugs were used specifically for migraine treatment (i.e., with indication restriction), compared to analyses without indication restrictions. This disparity in results according to indication restrictions likely reflects the prevalence of tinnitus as a symptom among migraine patients. Nevertheless, propranolol and topiramate still showed significant PRR and ROR values even in analyses not limited to migraine, making it difficult to rule out the possibility that these drugs could cause tinnitus. Notably, propranolol had a higher tinnitus risk index compared to serotonin receptor agonists or CGRP inhibitors, regardless of indication restrictions. While some reports suggest propranolol may induce tinnitus by affecting conductive tissues and increasing its intensity [34], others indicate it may alleviate certain types of tinnitus symptoms [35]. Although propranolol’s label does not mention the possibility of tinnitus induction, our study underscores the need for further research to explore its potential association with tinnitus occurrence, particularly in migraine patients.

Our study utilized the FAERS database, an open data source reflecting real-world conditions. While FAERS data provides the advantage of real-world evidence, it is important to acknowledge its limitations. Due to the nature of unrefined data, inaccuracies or errors may be present [17]; however, we believe that the analysis of large-scale data may mitigate these issues to some extent. Additionally, spontaneous adverse event reporting can result in duplicated cases, and causality cannot be definitively established due to the lack of verification. For instance, reports in the FAERS database are not always reviewed by a physician or audiologist; they are collected from a variety of sources, including both patients and healthcare professionals. Specific details regarding the characteristics of tinnitus are generally unavailable in the FAERS database. These include whether cases were acute or chronic, transient or progressive, their severity, timing of onset relative to drug administration, or resolution after discontinuation. Moreover, information about the type of medical intervention used to manage tinnitus or other contributing factors is also not reported. Therefore, while this study identifies potential associations between migraine treatments and tinnitus, further research, particularly clinical and observational studies, is essential to clarify these relationships. Despite these limitations, FAERS remains a crucial resource for identifying safety signals and generating hypotheses for further investigation, as it provides valuable insights into drug safety, including potential adverse events not captured in clinical trials [18,21].

## Conclusions

This study highlights potential associations between migraine treatments and the occurrence of tinnitus, offering preliminary insights that could inform future research and clinical decision-making. While our findings suggest that certain medications may be linked to a higher risk of tinnitus, we acknowledge that the small number of adverse event reports and the complexity of analyzing both acute and preventive treatments limit the strength of our conclusions. Our study does not provide definitive clinical recommendations but identifies areas where further research is needed to validate these associations and explore their clinical relevance. Clinicians should interpret these findings with caution and consider them as a starting point for deeper investigations into the safety profiles of migraine treatments. Ultimately, a more robust understanding of these potential risks will require larger studies and further exploration of the relationships between migraine, its treatments, and tinnitus.

## Supporting information

S1 Fig Inter-class analysis of migraine drugs without restriction for indication. Proportional reporting ratios (PRR) and reporting odds ratios (ROR), along with their respective 95% confidence intervals, contrast tinnitus cases associated with each migraine drug class against (a) CGRP inhibitors and (b) serotonin receptor agonists. CGRP stands for calcitonin gene-related peptide.
(PDF)
