# Supplementary material for: Evaluating the association between migraine treatments and tinnitus: Insights from the US Food and Drug Administration adverse event reporting system
Source: PLoS One. 2025 Aug 20;20(8):e0330493. doi: 10.1371/journal.pone.0330493 (PMC12367135; doi:10.1371/journal.pone.0330493)

S1 Fig. Inter-class analysis of migraine drugs without restriction for indication. Proportional reporting ratios (PRR) and reporting odds ratios (ROR), along with their respective 95% confidence intervals, contrast tinnitus cases associated with each migraine drug class against (a) CGRP inhibitors and (b) serotonin receptor agonists. CGRP stands for calcitonin gene-related peptide.

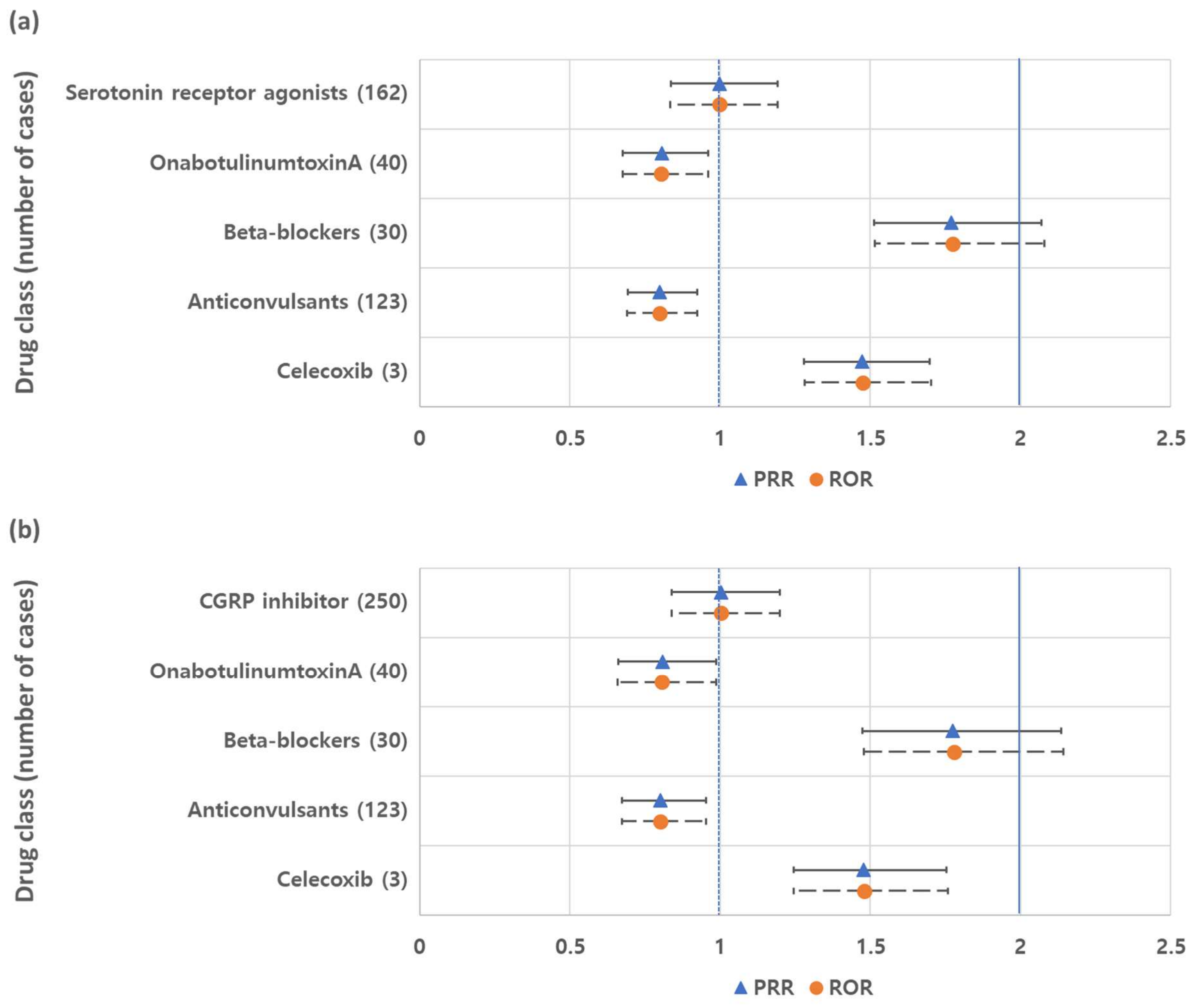

Supplement: S1 Fig — Proportional reporting ratios (PRR) and reporting odds ratios (ROR), along with their respective 95% confidence intervals, contrast tinnitus cases associated with each migraine drug class against (a) CGRP inhibitors and (b) serotonin receptor agonists. CGRP stands for calcitonin gene-related peptide. (PDF) [file pone.0330493.s001.pdf]
